# MMP9 Differentially Regulates Proteins Involved in Actin Polymerization and Cell Migration during TGF-β-Induced EMT in the Lens

**DOI:** 10.3390/ijms222111988

**Published:** 2021-11-05

**Authors:** Zi Zhen (Ginny) Liu, Aftab Taiyab, Judith A. West-Mays

**Affiliations:** Department of Pathology and Molecular Medicine, McMaster Health Sciences Center, Hamilton, ON L8N 3Z5, Canada; liuz15@mcmaster.ca (Z.Z.L.); taiyab@mcmaster.ca (A.T.)

**Keywords:** EMT, MMP9, TGF-β, lens, PCO, actin, cortactin, FAK, LIMK1, MLC2, MRTF-A

## Abstract

Fibrotic cataracts have been attributed to transforming growth factor-beta (TGF-β)-induced epithelial-to-mesenchymal transition (EMT). Using mouse knockout (KO) models, our laboratory has identified MMP9 as a crucial protein in the TGF-β-induced EMT process. In this study, we further revealed an absence of alpha-smooth muscle actin (αSMA) and filamentous-actin (F-actin) stress fibers in MMP9KO mouse lens epithelial cell explants (LECs). Expression analysis using NanoString revealed no marked differences in αSMA (*ACTA2*) and beta-actin (β-actin) (*ACTB*) mRNA between the lenses of TGF-β-overexpressing (TGF-βtg) mice and TGF-βtg mice on a MMP9KO background. We subsequently conducted a protein array that revealed differential regulation of proteins known to be involved in actin polymerization and cell migration in TGF-β-treated MMP9KO mouse LECs when compared to untreated controls. Immunofluorescence analyses using rat LECs and the novel MMP9-specific inhibitor, JNJ0966, revealed similar differential regulation of cortactin, FAK, LIMK1 and MLC2 as observed in the array. Finally, a reduction in the nuclear localization of MRTF-A, a master regulator of cytoskeletal remodeling during EMT, was observed in rat LECs co-treated with JNJ0966 and TGF-β. In conclusion, MMP9 deficiency results in differential regulation of proteins involved in actin polymerization and cell migration, and this in turn prevents TGF-β-induced EMT in the lens.

## 1. Introduction

Cataract is the leading cause of blindness, affecting 94 million people around the world [1], and the most common resolution is cataract surgery [2]. Although this procedure is generally deemed safe, posterior capsular opacification (PCO), a fibrotic cataract, can develop in up to 50% of patients post-surgery [3]. PCO is one of the two major forms of fibrotic cataracts that are implicated by transforming growth factor-beta (TGF-β)-induced epithelial-to-mesenchymal transition (EMT) [2]. PCO occurs after the ocular lens experiences tremendous stress during cataract surgery, and although most of the lens epithelial cells on the anterior lens capsule are removed, some persistent cells remain in the germinative and transitional zones [4,5]. Increased levels of TGF-β post-surgery stimulate these cells to undergo EMT, which results in the migration of the lens epithelial cells to the posterior capsule, and causes capsular wrinkling, deposition of aberrant extracellular proteins, and multilayering of cells at the posterior lens [4,6].

TGF-β is a group of multifunctional cytokines that play key roles in embryogenesis, cell differentiation, wound healing, cell adhesion and apoptosis [7,8]. It is also a potent inducer of pathological conditions including fibrosis and cancer [9,10]. Endogenous mature TGF-β in the aqueous humor is heterotetrametric in its latent form and consists of an active TGF-β homodimer attached to its latency-associated protein (LAP) [10,11]. The LAP is cleaved upon activation [2,11] and active TGF-β binds to TGF-β receptors to initiate various intracellular pathways [8]. Disturbances in the negative regulation and termination of TGF-β signaling result in pathological conditions including fibrosis and cancer [8,9]. As true in other systems such as the kidney and the lung, the lens also shows the transdifferentiation of cuboidal epithelial cells into mesenchymal myofibroblasts during TGF-β-induced EMT [9]. This process involves the loss of epithelial characteristics such as marginalized epithelial cadherin (E-cadherin) and the gain of mesenchymal characteristics such as alpha-smooth muscle actin (αSMA) and filamentous actin (F-actin) stress fibers [9,12,13].

TGF-β induced fibrosis proceeds via Smad-dependent and Smad-independent pathways [14]. The most characterized TGF-β signaling pathway is the canonical Smad pathway, which induces the expression of EMT-associated genes after the nuclear translocation of the Smad2/3-Smad4 complex from the cytoplasm [8]. There are numerous TGF-β-induced Smad-independent pathways, including the p38, ERK1/2, and PI3K and Rho/ROCK pathways [14,15], but these pathways have also been shown to interact with Smad-dependent pathways in the lens [13]. Using the LEC model, we and others have demonstrated the contribution of both canonical and non-canonical TGF-β signaling pathways including the Smad, β-catenin and Rho/ROCK pathways during TGF-β-induced EMT of the lens [13,15,16,17,18].

Matrix metalloproteinases (MMPs) are a family of zinc-dependent proteases that degrade the extracellular matrix, and are involved in processes such as embryogenesis, wound healing and fibrosis [19]. MMP2 and MMP9 are associated with TGF-β-induced EMT since they are known to activate latent TGF-β in the aqueous humor, and active TGF-β can in turn upregulate MMP2 and MMP9 [2]. Our laboratory has shown that co-treatment with a MMP2/9-specific inhibitor and TGF-β reduced EMT and ASC in the ocular lens [20]. Additional in vivo and ex vivo MMP9 knock-out (KO) mouse studies from our laboratory demonstrated that MMP9 is essential for TGF-β-induced EMT as the lack of MMP9 conferred resistance against EMT along with the absence of the expression of the key EMT marker, αSMA [12]. Interestingly, MMP9 deficiency alone results in an altered cytoskeleton in comparison to MMP2KO and wildtype mice LECs [12]. In the current manuscript, we sought to further understand how the lack of MMP9 causes cytoskeletal modifications. More specifically, we performed a comprehensive examination of the proteins responsible for the cytoskeletal changes in MMP9 deficient models and aimed to identify the roles of these proteins during TGF-β- induced EMT in LECs.

## 2. Results

### 2.1. Absence of MMP9 Leads to Defects in TGF-β-Induced Actin Polymerization and Differential Expression of αSMA

**Figure 1:**
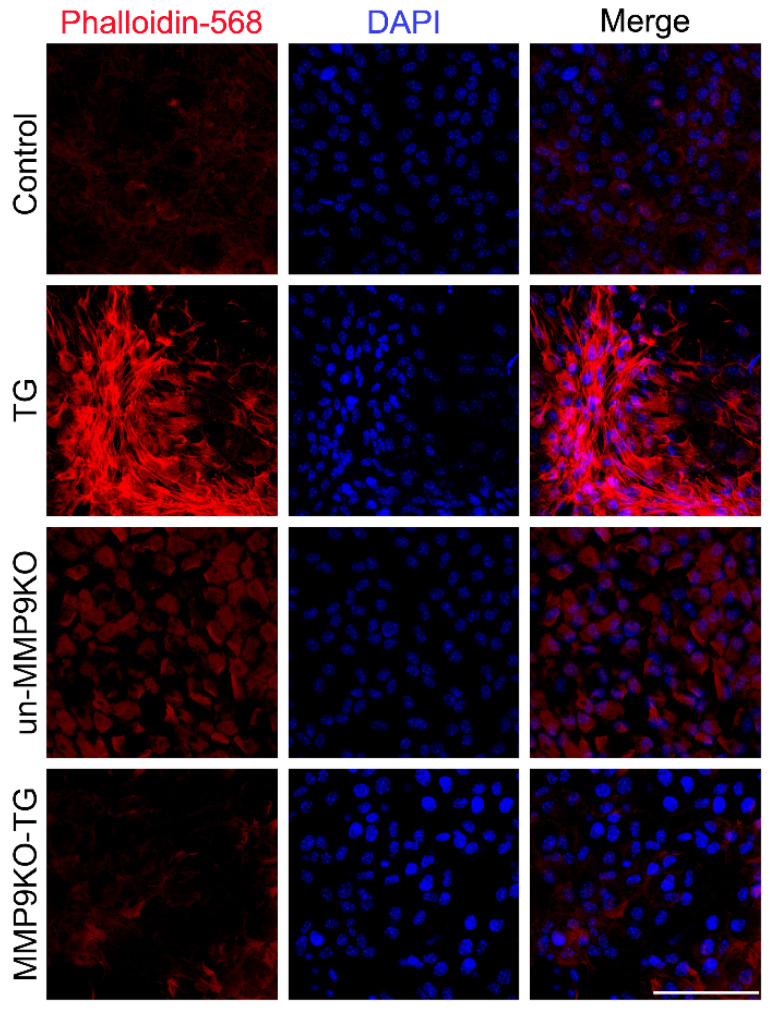
F-actin polymerization upon TGF-β stimulation. LEC explants from wildtype mice were left untreated (control) or treated with 500 pg/mL TGF-β for 72 h (TG). LEC explants from MMP9KO mice were left untreated (un-MMP9KO) or treated with 500 pg/mL TGF-β for 72 h (MMP9KO-TG) (*n* = 3 independent experiments, where *n* ≥ 3 LECs per treatment were used for each experiment). Paraformaldehyde (PFA) fixed explants were stained for F-actin using phalloidin-568. Images were acquired using Leica DM6 fluorescence microscope at 40×. Scale bar, 100 µm.

MMPs have been shown to play a critical role in cytoskeletal reorganization as the absence of MMP9 has been correlated with a lack of F-actin in astrocytes [21]. We have also shown that LECs from MMP9KO mouse eyes were unable to polymerize the inducible form of actin, αSMA, in the presence of TGF-β [12]. To further pursue this, we examined whether LECs lacking MMP9 showed an inability to polymerize F-actin as well when stimulated with TGF-β. Indeed, polymerized actin, as visualized by staining with phalloidin, was absent in the TGF-β treated MMP9KO mouse LECs when compared to TGF-β treated wildtype mouse LECs (Figure 1).

**Figure 2:**
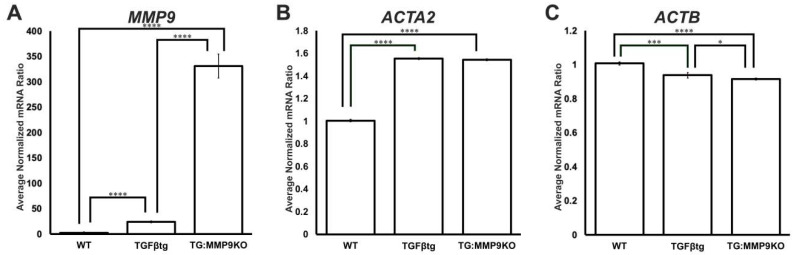
Graphs showing average expression of *ACTA2, ACTB* and *MMP9* during NanoString analysis. Lenses were obtained from wildtype (WT) (*n* = 4 experiments, where *n* = 3 lenses per experiment), TGF-β overexpressing (TGFβtg) (*n* = 4 experiments, where *n* = 3 lenses per experiment) and TGFβtg on the MMP9KO background (TG: MMP9KO) (*n* = 4 independent experiment, where *n* = 3 lenses per experiment) mice. The NanoString nCounter gene expression system was used to quantify the expression of *MMP9* (**A**)*, ACTA2* (**B**) and *ACTB* (**C**). The data was normalized to total RNA count, and the normalized mRNA ratios were calculated by referencing to one set of WT. Error bars indicate ± standard deviation of the average normalized mRNA ratios (* *p* < 0.05; *** *p* < 0.001; **** *p* < 0.0001).

In order to determine whether MMP9 plays a role in regulating the gene expression of actin, and therefore resulting in a lack of αSMA and F-actin, a NanoString analysis was performed to quantify the number of mRNA transcripts for αSMA (*ACTA2*) and β-actin (*ACTB*). LECs from wildtype mice, transgenic mice overexpressing TGF-β (TGFβtg) and TGFβtg on MMP9KO background (TG:MMP9KO) (Figure 2) were used to perform the experiment four times. Our MMP9KO mice could still express the mRNA for MMP9, but the mRNA cannot code for a functional protein as a portion of exon 2 and all of intron 2 were replaced with a *pgk-neo* gene cassette [12]. Therefore, in order to ensure that our transgenic TG:MMP9KO mouse model was functional, MMP9 expression was also analyzed as a positive control during NanoString analysis because TGF-β is known to upregulate MMP9 expression [22]. A 12-fold upregulation in MMP9 expression was observed in TGFβtg LECs when compared to wildtype LECs (Figure 2A; **** *p* < 0.0001). Furthermore, a 150-fold increase in MMP9 expression was observed in the TG:MMP9KO versus wildtype LECs when compared to a 13-fold increase in MMP9 expression between TG:MMP9KO and TGFβtg LECs (Figure 2A; **** *p* < 0.0001). There was a significant 1.5-fold increase in αSMA (*ACTA2*) expression in TGFβtg LECs (1.55 ± 0.01) and TG:MMP9KO LECs (1.54 ± 0.01) when compared to wildtype LECs (1.01 ± 0.01) (Figure 2B; **** *p* < 0.0001). However, *ACTA2* levels were not significantly different between TGFβtg and TG:MMP9KO LECs (Figure 2B; *p* = 0.55). In addition, there was no marked difference in actin expression (*ACTB*) between wildtype and TGFβtg LECs (Figure 2B; *** *p* < 0.001), and between wildtype and TG:MMP9KO LECs (Figure 2C; **** *p* < 0.0001). *ACTB* expression was not notably different between TGFβtg and MMP9KO-TG LECs (Figure 2C; * *p* < 0.05). Although αSMA and F-actin polymerization were not observed in MMP9KO mouse LECs that were stimulated with an exogenous source of TGF-β (Figure 1) [12], no marked difference was observed in *ACTA2* and *ACTB* expression between TGFβtg and TG:MMP9KO mouse LECs (Figure 2B,C). Hence, we proposed that components of the actin polymerization machinery may be inactive as a result of the lack of MMP9.

### 2.2. MMP9 Differentially Regulates Cytoskeletal Components Involved in Actin Polymerization 

**Figure 3:**
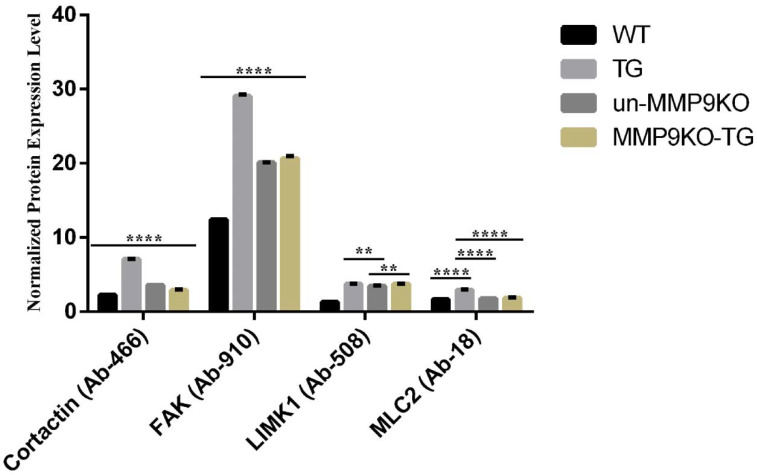
Graphs showing the average signal of protein expression for selected proteins. Cytoskeletal protein array analysis was conducted using LEC explants from wildtype (control) and MMP9KO mice were left untreated (un-MMP9KO) or with treated with 500 pg/mL TGF-β for 72 h (MMP9KO-TG). Cortactin, focal adhesion kinase (FAK), lim-domain kinase-1 (LIMK1) and myosin light chain-2 (MLC2) were analyzed. Data was normalized to the median GAPDH and then averaged. A 2-way ANOVA with multiple comparisons was performed and the data was graphed using Graphpad Prism. Error bars indicate ± standard deviation (*n* = 3 independent experiments, where 10 µg of protein per treatment was used for each experiment; ** *p* < 0.01 **** *p* < 0.0001). For Cortactin and FAK, *p* < 0.0001 across all treatment groups i.e., WT vs. TG or un-MMP9KO or MMP9KO-TG, TG vs. un-MMP9KO or MMP9KO-TG and un-MMP9KO vs. MMP9KO-TG. For LIMK1, *p* < 0.0001 WT vs. other treatments; *p* < 0.01 TG vs. un-MMP9KO and un-MMP9KO vs. MMP9KO-TG. For MLC2, *p* < 0.0001 WT vs. TG, TG vs. un-MMP9KO and TG vs. MMP9KO-TG.

To demonstrate the role of MMP9 in modulating the expression and activation of proteins required during actin polymerization and reorganization, we performed a cytoskeletal protein array (Fullmoon Biosystems) using untreated and TGF-β-treated wildtype and MMP9KO LECs. Numerous proteins were observed to be differentially expressed and phosphorylated upon TGF-β stimulation in MMP9KO LECs (Table 1). The protein array showed that nine proteins were upregulated by 1.26 to 3.11-fold in TGF-β treated wildtype LECs (TG) when compared to untreated wildtype LECs (control), but no marked upregulations were observed between TGF-β treated MMP9KO (MMP9KO-TG) and untreated MMP9KO (un-MMP9KO) LECs (Table 1). Based on fold-changes of 1.5 or greater between TG and control LECs, cortactin, focal adhesion kinase (FAK), lim-domain kinase-1 (LIMK1) and myosin light chain-2 (MLC2) were considered for further analyses. A 3.11-fold increase in cortactin levels, known to be critical for F-actin polymerization and branching [23], was observed between TG (7.11 ± 0.04) and control (2.29 ± 0.0005) LECs (Table 1 and Figure 3; **** *p <* 0.0001). However, no marked difference in cortactin levels was observed in MMP9KO-TG LECs (3.63 ± 0.002) when compared to untreated un-MMP9KO LECs (2.90 ± 0.07) (Table 1 and Figure 3; **** *p <* 0.0001). Another important protein that showed a notable difference in expression was FAK, a major component of focal adhesion complexes that has been shown to play critical roles in actin cytoskeletal remodeling, stress fiber formation and cell migration [24]. The expression of FAK was upregulated in TG LECs (29.08 ± 0.2) by 2.34-fold in comparison to control LECs (12.41 ± 0.05) (Table 1 and Figure 3; **** *p* < 0.0001). The MMP9KO-TG LECs (20.69 ± 0.3) failed to show a notable upregulation of FAK when compared to un-MMP9KO LECs (20.11 ± 0.02) (Table 1 and Figure 3; **** *p* < 0.0001). In addition, we observed a 1.27-fold increase in phosphorylated, and therefore activated, FAK (pFAK) in TG LECs when compared to control LECs, but no marked upregulation of pFAK was observed between MMP9KO-TG and un-MMP9KO LECs (Table 1). Another protein that was differentially regulated was LIMK1, which is a major regulator of actin dynamics by phosphorylating and inhibiting the key actin-depolymerizing protein, cofilin [25]. The expression of LIMK1 was observed to be upregulated by 2.85-fold in TG LECs (3.75 ± 0.03) when compared to control LECs (1.31 ± 0.001) (Table 1 and Figure 3; **** *p* < 0.0001), but no marked upregulation was observed between MMP9KO-TG (3.76 ± 0.04) and un-MMP9KO (3.49 ± 0.01) LECs (Table 1 and Figure 3; ** *p* < 0.01). Furthermore, the phosphorylation, and therefore activation, of LIMK1 (pLIMK1) was 1.26-fold higher in TG LECs when compared to control LECs, but this upregulation was also not observed between MMP9KO-TG and un-MMP9KO LECs (Table 1). MLC2 is crucial for myosin-actin cross-bridge cycling and contractility [26], and therefore the upregulation and activation of MLC2 indicate increased contractility of LECs. We observed a 1.74-fold increase in MLC2 levels between TG (2.95 ± 0.02) and control LECs (1.69 ± 0.004) (Table 1 and Figure 3; **** *p* < 0.0001), but this upregulation was not observed between MMP9KO-TG (1.89 ± 0.03) and un-MMP9KO LECs (1.79 ± 0.002) (Table 1 and Figure 3; not significant). We also observed a 1.54-fold increase in phosphorylated, and therefore activated, MLC2 (pMLC2) between TG and control LECs, but no marked difference in pMLC2 was observed between MMP9KO-TG and un-MMP9KO LECs (Table 1).

### 2.3. A MMP9-Specific Inhibitor of Activation Prevented EMT in Rat LECs by Differentially Regulating Cytoskeletal Components Involved in Actin Polymerization

To validate the observed protein levels from the protein array, and to investigate the localization of the proteins, immunofluorescence analysis was carried out using rat LEC explants and a MMP9-specific allosteric inhibitor of activation, JNJ0966 [27]. This inhibitor has no effect on the catalytic activities of other MMPs such as MMP1 and MMP14, and it did not inhibit the activation of MMP2, which has a similar activation site as MMP9 [27]. The efficacy of the inhibitor behaves in a dose-dependent manner [27], and we determined that a 2-h pre-treatment with 20 µM of JNJ0966 could prevent the elongation of rat LECs that have been exposed to 6 ng/mL of TGF-β for 48 h. Immunofluorescence analysis was conducted to further confirm the efficacy of JNJ0966.

**Figure 4:**
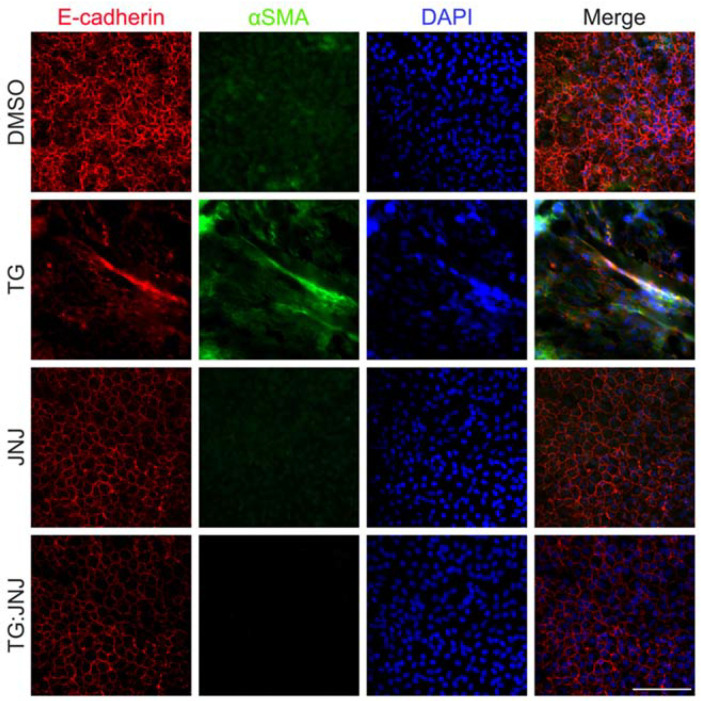
Localization and expression of E-cadherin and αSMA upon MMP9 inhibition. Rat LEC explants were treated with DMSO, with 6 ng/mL TGF-β for 48 h (TG), with the MMP9-specific inhibitor, 20 µM JNJ0966 (JNJ) for 48 h, or pretreated with 20 µM JNJ0966 for 2 h followed by 6 ng/mL TGF-β for 48 h (TG:JNJ) (*n* = 3 independent experiments, where *n* ≥ 3 LECs per treatment were used for each experiment). Paraformaldehyde (PFA) fixed explants were stained for E-cadherin (red) and αSMA (green), and mounted with DAPI to visualize the nuclei. Images were acquired using Leica DM6 fluorescence microscope at 40×. Scale bar, 100 µm.

Figure 4 shows increased αSMA expression in rat LECs treated with TGF-β (TG) when compared to rat LECs treated with <5% of dimethyl sulfoxide (DMSO control), which was the solvent for JNJ0966. More importantly, LECs that were only treated with JNJ0966 (JNJ) and LECs that were pretreated with JNJ and then treated with TGF-β (TG:JNJ) showed similar αSMA immunofluorescence staining as DMSO controls (Figure 4). To provide additional assurance that JNJ0966 inhibits MMP9 and prevents EMT, the presence of E-cadherin was also analyzed. As expected, E-cadherin was present and localized to cell margins in DMSO control, JNJ and TG:JNJ LECs, but E-cadherin was reduced and delocalized in TG LECs (Figure 4). It is important to point out that in the TG treated explants the number of cell bodies visible in the images obtained appeared reduced compared to other treatment groups and this is mainly due to the fact that myofibroblasts (after EMT has been induced) exhibit a larger cell volume, resulting in fewer cells being captured in any given image. As outlined in our previously published work, TG treatment of rat lens explants also caused an increase in cell death, but this was found to be very negligible [28].

**Figure 5:**
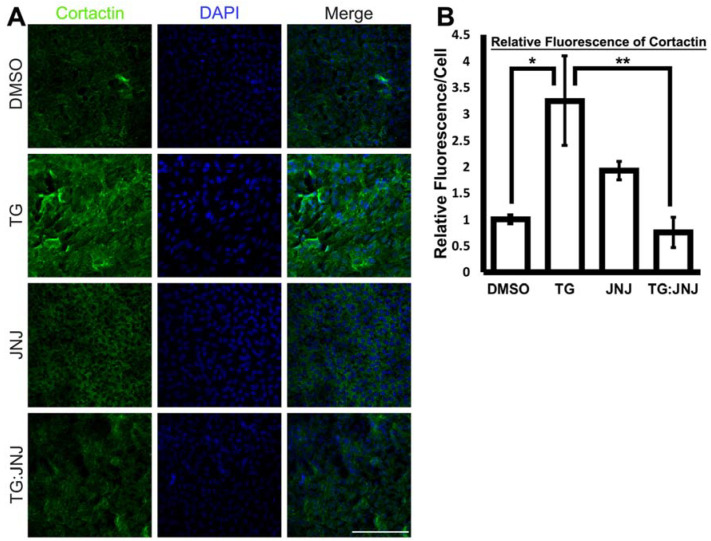
Expression of cortactin upon MMP9 inhibition. Rat LEC explants were treated with DMSO, with 6 ng/mL TGF-β for 48 h (TG), with the MMP9-specific inhibitor, 20 µM JNJ0966 (JNJ) for 48 h, or pretreated with 20 µM JNJ0966 for 2 h followed by 6 ng/mL TGF-β for 48 h (TG:JNJ) (*n* = 3 independent experiments, where *n* ≥ 3 LECs per treatment were used for each experiment). (**A**) Paraformaldehyde (PFA) fixed explants were stained for cortactin (green) and mounted with DAPI to visualize the nuclei. Images were acquired using Leica DM6 fluorescence microscope at 40×. Scale bar, 100 µm. (**B**) Graph showing mean cortactin fluorescence per cell relative to LECs treated with DMSO. Fluorescence per cell was acquired using Image J (*n* ≥ 3 LECs per treatment). Error bars indicate ± SEM of the relative mean fluorescence (One-Way ANOVA shows ** *p* < 0.01; Tukey’s Test shows * *p* < 0.05 between DMSO control and TG LECs and ** *p* < 0.01 between TG and TG:JNJ LECs).

After confirming that the treatment with JNJ0966 prevented TGF-β-induced EMT in rat LECs, the expression and localization of the proteins of interest were validated and assessed using immunohistochemistry. Cortactin was upregulated in TG LECs in comparison to the DMSO control, and immunofluorescence staining for cortactin in JNJ and TG:JNJ LECs resembled that of the DMSO control LECs (Figure 5A). The graph shows a threefold increase in cortactin in TG LECs relative to DMSO control LECs (Figure 5B; * *p* < 0.05). The mean fluorescence values of cortactin for TG:JNJ LECs was observed to be similar to the DMSO control LECs thereby showing the involvement of MMP9 in modulation of cortactin expression (Figure 5B).

**Figure 6:**
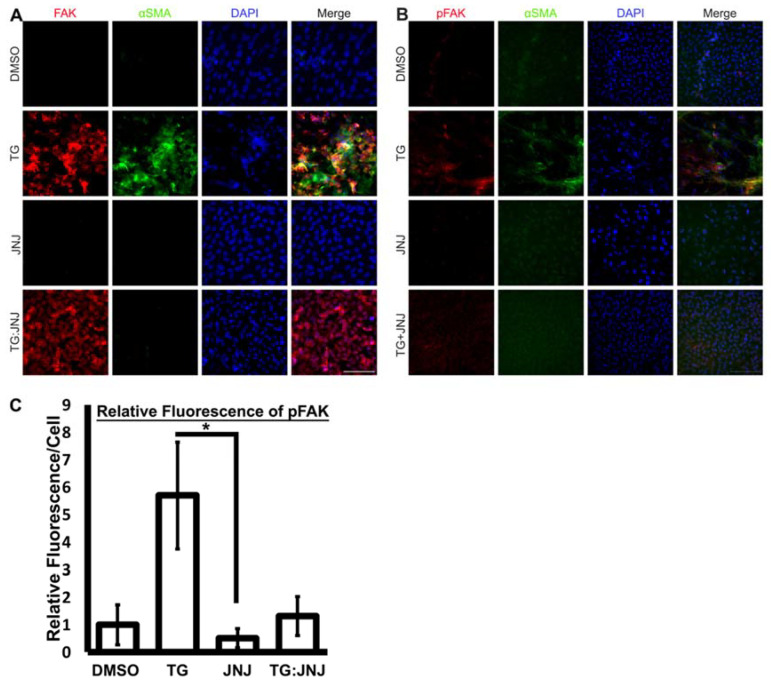
Expression of focal adhesion kinase (FAK) and phosphorylated FAK at Tyr397 (pFAK) upon MMP9 inhibition. Rat LEC explants were treated with DMSO, with 6 ng/mL TGF-β for 48 h (TG), with 20 µM JNJ0966 (JNJ) for 48 h, or pretreated with 20 µM JNJ0966 for 2 h followed by 6 ng/mL TGF-β for 48 h (TG:JNJ) (*n* = 3 independent experiments, where *n* ≥ 3 LECS per treatment were used for each experiment). Paraformaldehyde (PFA) fixed explants were stained for FAK (**A**; red) and pFAK (**B**; red) and αSMA (**A**,**B**; green), and mounted with DAPI to visualize the nuclei. Images were acquired using Leica DM6 fluorescence microscope at 40×. Scale bar, 100 µm. (**C**) Graph showing mean pFAK fluorescence per cell relative to LECs treated with DMSO. Fluorescence per cell was acquired using Image J (*n* = 3 LECs per treatment). Error bars indicate ± SEM of the relative mean fluorescence (One-Way ANOVA shows * *p* < 0.05; Tukey’s Test shows * *p* < 0.05 between TG LECs and JNJ LECs).

**Figure 7:**
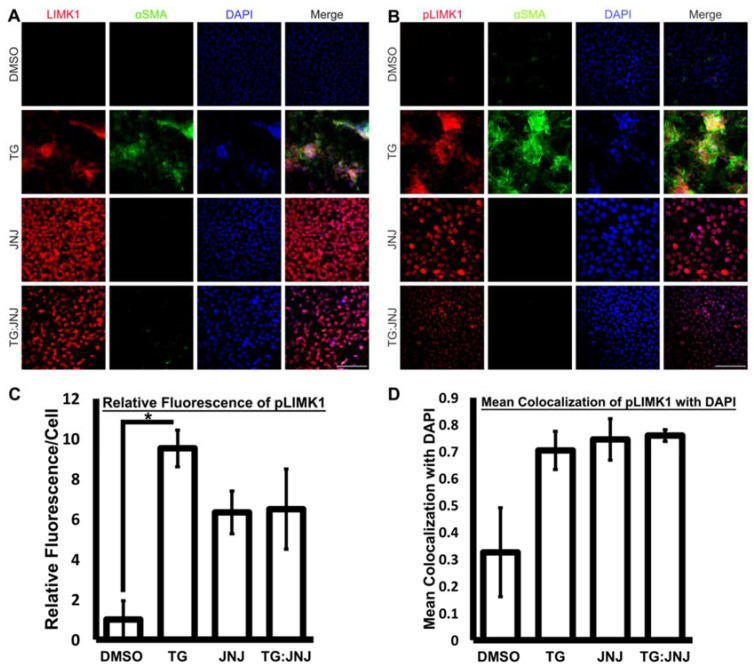
Localization of overall lim-domain kinase-1(LIMK1) and phosphorylated LIMK1 at Thr508 (pLIMK1) upon MMP9 inhibition. Rat LEC explants were treated with <5% DMSO, with 6 ng/mL TGF-β for 48 h (TG), with 20 µM JNJ0966 (JNJ) for 48 h, or pretreated with 20 µM JNJ0966 for 2 h followed by 6 ng TGF-β for 48 h (TG:JNJ) (*n* = 3 independent experiments, where *n* ≥ 3 LECs per treatment were used for each experiment). Paraformaldehyde (PFA) fixed explants were stained for LIMK1 (**A**), pLIMK1 (**B**) and αSMA, and mounted with DAPI to visualize the nuclei. Images were acquired using Leica DM6 fluorescence microscope at 40×. Scale bar, 100 µm. (**C**) Graph showing mean pLIMK1 fluorescence per cell relative to LECs treated with DMSO. Fluorescence per cell was acquired using Image J (*n* ≥ 3 LECs per treatment). Error bars indicate ± SEM of the relative mean fluorescence (One-Way ANOVA shows * *p* < 0.05; Tukey’s Test shows * *p* < 0.05 between DMSO control and TG LECs). (**D**) Graph showing Manders’ coefficient for fraction of pLIMK1 colocalizing with DAPI (*n* ≥ 3 LECs per treatment). Colocalization was detected using Image J. Error bars indicate ± SEM (One-Way ANOVA shows * *p* < 0.05; Tukey’s Test indicates no significance between the four treatment groups).

**Figure 8:**
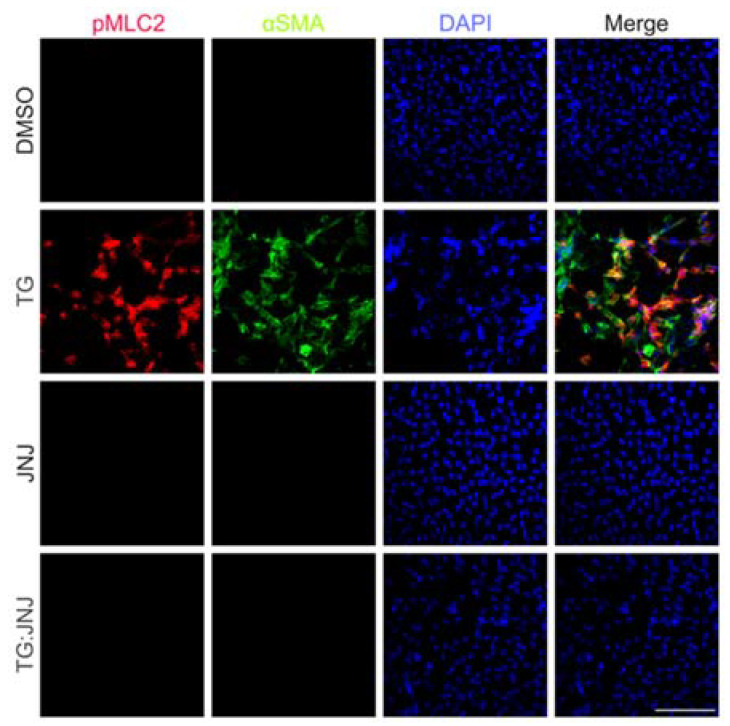
Localization of phosphorylated myosin light chain-2 at Ser18 (pMLC2) upon MMP9 inhibition. Rat LEC explants were treated with <5% DMSO, with 6 ng/mL TGF-β for 48 h (TG), with 20 µM JNJ0966 (JNJ) for 48 h, or pretreated with 20 µM JNJ0966 for 2 h followed by 6 ng/mL TGF-β for 48 h (TG:JNJ) (*n* = 3 independent experiments where *n* ≥ 3 LECs per treatment were used for each experiment). Paraformaldehyde (PFA) fixed explants were stained for pMLC2 (red) and αSMA (green), and mounted with DAPI to visualize the nuclei. Images were acquired using Leica DM6 fluorescence microscope at 40×. Scale bar, 100 µm.

Furthermore, during immunofluorescence analysis, FAK and αSMA were upregulated in TG LECs in comparison to DMSO control LECs (Figure 6A). However, FAK expression was also upregulated in TG:JNJ LECS in comparison to JNJ LECs, but no noticeable αSMA expression was observed in either TG:JNJ or JNJ LECs (Figure 6A). Since TGF-β upregulated overall FAK in MMP9-inhibited LECs, the autophosphorylation of FAK at Tyr397 (pFAK), which indicates the protein’s activation [29], was further analyzed. Immunofluorescence analysis indicated that pFAK and αSMA levels were elevated in TG LECs in comparison to DMSO control, JNJ and TG:JNJ LECs (Figure 6B). We further analyzed the fluorescence of pFAK using ImageJ. Figure 6C shows ~5.6-fold increase in pFAK in TG LECs compared to DMSO control LECs. This TGF-β-induced increase in pFAK was inhibited in TG:JNJ LECs in which the fluorescence levels was observed to be similar DMSO control LECs (Figure 6C). The next protein that was analyzed was LIMK1, which was upregulated in TG, JNJ and TG:JNJ LECs when compared to DMSO control LECs (Figure 7A). However, LIMK1 appeared to colocalize with DAPI in JNJ and TG:JNJ LECs, and αSMA was not observed, when compared to more diffuse localizations of LIMK1, and αSMA expression, in TG LECs (Figure 7A). Since no noticeable difference in LIMK1 expression was detected between JNJ and TG:JNJ LECs, additional experiments were performed to analyze the phosphorylation of LIMK1 at Thr508 (pLIMK1), which indicates the protein’s activation [30]. Immunofluorescence staining from Figure 7B shows upregulations of pLIMK1 in TG and TG:JNJ LECs when compared to DMSO control and JNJ LECs respectively, but αSMA was observed to be upregulated in TG LECs only. The graph in Figure 7C clearly shows ~9-fold increase in pLIMK1 in TG LECs that decreased to ~6-fold upon inhibition of MMP9 (TG:JNJ LECs) when compared to the DMSO control LECs. Furthermore, the localization of pLIMK1 appeared to be cytoplasmic and nuclear in TG LECs, but pLIMK1 colocalized with DAPI in TG:JNJ LECs (Figure 7B). Our colocalization analysis reveals an increase in nuclear localization of pLIMK1 in TG LECs (~3-fold; Figure 7D) that did not decrease upon inhibition of MMP9 (TG:JNJ LECs; Figure 7D). It was noted from the protein array that the average protein level of phosphorylated MLC2 was greater than that of overall MLC2 in un-MMP9KO mouse LECs (2.95 versus 1.79 respectively) and MMP9KO-TG mouse LECs (2.14 versus 1.89 respectively). Therefore, the phosphorylated form of MLC2 (pMLC2) was selected for further validation. Immunofluorescence staining for pMLC2 at Ser18 showed an upregulation of pMLC2 and αSMA in TG LECs when compared to DMSO control, JNJ and TG:JNJ LECs (Figure 8).

### 2.4. MMP9 Deficiency Notably Reduced MRTF-A Translocation to the Nucleus 

**Figure 9:**
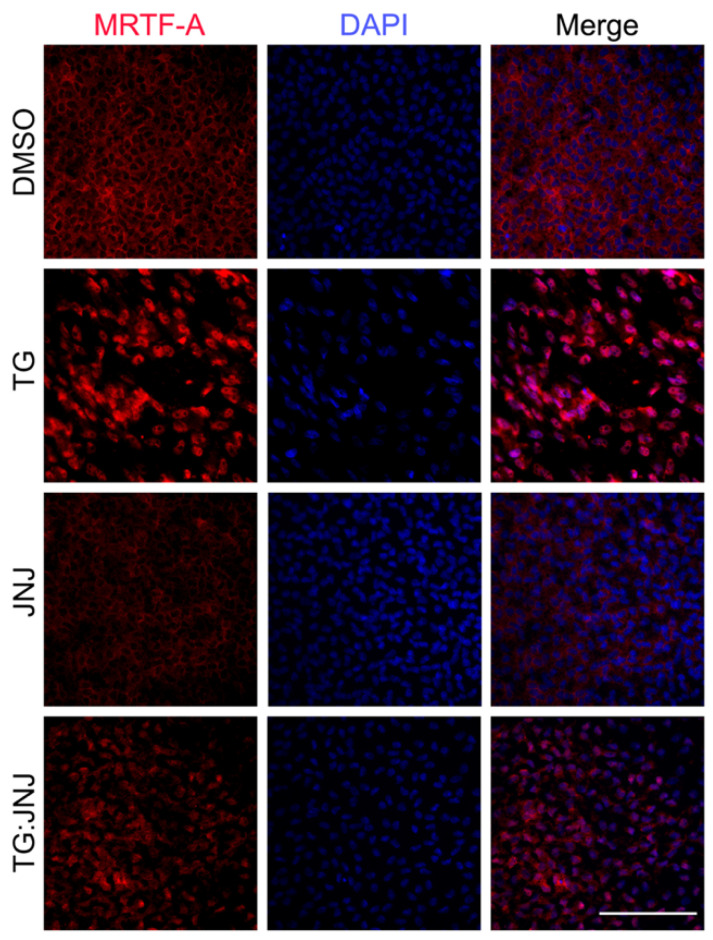
Localization of myocardin-related transcription factor-A (MRTF-A) upon MMP9 inhibition. Rat LEC explants were treated with <5% DMSO, with 6 ng/mL TGF-β for 48 h (TG), with 20 µM JNJ0966 (JNJ) for 48 h, or pretreated with 20 µM JNJ0966 for 2 h followed by 6 ng/mL TGF-β for 48 h (TG:JNJ) (*n* = 3 independent experiments, where *n* ≥ 3 LECs per treatment were used for each experiment). Paraformaldehyde (PFA) fixed explants were stained for MRTF-A (red) and mounted with DAPI to visualize the nuclei. Images were acquired using Leica DM6 fluorescence microscope at 40×. Scale bar 100 µm.

The actin/myocardin-related response factor (MRTF-A)/serum response factor (SRF) circuit plays a crucial role in mediating cytoskeletal modifications and stress fiber formation during TGF-β implicated fibrosis [31,32]. Since the actin polymerization machinery was inactive in MMP9-inhibited explants, we proposed that the Rho/ROCK pathway and the downstream dissociation of G-actin from MRTF-A to provide actin monomers for polymerization were affected [33]. The lack of dissociation of G-actin from MRTF-A would result in reduced MRTF-A translocation to the nucleus [33]. There would thus be less MRFT-A to interact with SRF in the nucleus to upregulate stress fiber formation and cytoskeletal remodeling [31]. Hence, immunofluorescence staining for MRTF-A was performed by using rat LECs and JNJ0966. Figure 9 shows nuclear localization of MRTF-A in TG LECs, cytoplasmic localization of MRTF-A in DMSO control and JNJ LECs, and mostly cytoplasmic localization of MRTF-A in TG:JNJ LECs.

## 3. Discussion

Understanding the mechanisms by which TGF-β induces EMT, a major cause of fibrosis, is crucial for the development of therapeutics for preventing this irreversible condition. Ex vivo experiments using mouse LECs from our laboratory have shown that the absence of MMP9 can prevent TGF-β-induced EMT [12]. In addition, in vivo studies showed that MMP9 deficiency conferred resistance against TGF-β-induced EMT when TGF-β was overexpressed in the lens during embryogenesis [12]. Our further studies revealed modifications in the cytoskeleton of the LECs of MMP9KO mice [12]. Therefore, using the LEC explant model, the present study aimed to investigate the cytoskeletal components that may be modified due to MMP9 deficiency and the roles these proteins play in TGF-β-induced EMT. Immunofluorescence analyses showed that there was a lack of TGF-β-induced stress fiber formation as F-actin polymerization (Figure 1) and αSMA expression were not observed in MMP9KO mouse LECs that were treated with TGF-β [12]. To further confirm these observations, a NanoString analysis was performed which showed no significant differences in the mRNA expression of αSMA (*ACTA2*) and β-actin (*ACTB*), between TGFβtg mouse lenses when compared to TG:MMP9KO mouse lenses (Figure 2B,C). Since actin mRNA expression was not observed to be affected, but F-actin and αSMA protein expressions were absent in MMP9KO mouse LECs when stimulated with TGF-β, we surmised that the actin polymerization machinery may be differentially regulated at the protein level as a result of the MMP9 deficiency.

The protein array showed an upregulation of cortactin between TG mouse and control mouse LECs, but this upregulation was not observed between un-MMP9KO and MMP9KO-TG LECs (Figure 3 and Figure 4A). Immunofluorescence staining for cortactin (Figure 5) agreed with the protein array result, and revealed perinuclear localization of cortactin in DMSO control, JNJ and TG:JNJ rat LECs with cuboidal-shaped cells, whereas cortactin staining appeared straited and the cells were elongated in TG rat LECs (Figure 5). Cortactin plays an important role in F-actin formation by binding with the actin associated protein (Arp) 2/3 complex to stimulate actin polymerization and branching [23,34,35]. Cortactin also has a role in F-actin polymer stabilization by binding to newly added actin monomers with ATP or ADP-Pi, and cortical association of cortactin with F-actin is associated with cell migration [23,34]. Therefore, the lack of observed F-actin polymers in TGF-β treated MMP9 deficient LECs could be due to the lack of cortactin upregulation thereby affecting actin polymerization, branching and stabilization.

While cortactin binds to newly added actin monomers, cofilin, an actin depolymerizing protein, binds to older actin monomers with ADP. LIMK1, which can be phosphorylated and thus activated by Rho/ROCK signaling at Thr508, is a crucial regulator of this depolymerization process by phosphorylating and inhibiting cofilin [23,25]. Therefore, increased activation of LIMK1 is responsible for F-actin maintenance during TGF-β-induced EMT. The protein array showed an upregulation of LIMK1 between TG and control mouse LECs, and no marked upregulation between un-MMP9KO and MMP9KO-TG mouse LECs (Table 1 and Figure 3). However, there was a notable upregulation of LIMK1 in un-MMP9KO when compared to control mouse LECs (Figure 3). Immunofluorescence analyses for both LIMK1 and activated LIMK1 revealed similar patterns of upregulation as the protein array (Figure 7). More interestingly, the localizations of LIMK1 and activated LIMK1 were nuclear, as LIMK1 appeared to colocalize with DAPI, in JNJ and TG:JNJ rat LECs in comparison to cytoplasmic and nuclear localization of LIMK1 and activated LIMK1 in TG rat LECs (Figure 7). Investigations from other laboratories have shown that LIMK1 can shuttle between the nucleus and cytoplasm, and that both nuclear and cytoplasmic LIMK1 could implicate breast cancer progression by activating the FAK/paxillin/Src/AKT/Erk pathway and therefore increasing FAK activation at focal adhesions [36,37]. In addition, recent investigations suggest that nuclear LIMK1 can bind to the promoter of c-myc to upregulate c-myc transcription in hepatic carcinomas [38], and c-myc overexpression has been shown to promote fibrogenesis in hepatic stellate cells [39]. However, further investigations are required to study the role of LIMK1 in the nucleus during TGF-β-induced fibrosis in LECs.

FAK was also observed to colocalize with DAPI, indicating nuclear localization, in TG:JNJ rat LECs during immunohistochemistry (Figure 6A). In addition to being localized in the nucleus, FAK was also upregulated in TG:JNJ LECs when compared to JNJ LECs, which differed from the upregulation patterns observed from the protein array (Figure 3 and Figure 4B). The function of FAK in the nucleus is unclear, but recent investigations have shown the potential for FAK to be a co-transcriptional regulator during cancer progression [40], which differs from the traditional understanding of its roles in focal adhesions [24]. However, the upregulation of FAK and its role in the nucleus require further investigation in the lens and other fibrotic models. Although FAK was notably upregulated in TG:JNJ rat LECs, the phosphorylated (at Tyr397), and therefore active, form of the protein [28], pFAK, was not upregulated in TG:JNJ rat LECs when compared to JNJ and DMSO control rat LECs (Figure 6B,C). These immunofluorescence analyses are in agreement with the protein array results where pFAK was only upregulated in TG mouse LECs when compared to control mouse LECs (Table 1). Therefore, MMP9 appears to regulate the activation, not the expression of FAK during TGF-β-induced EMT. FAK is activated via autophosphorylation at Tyr397 due to integrin clustering, which can occur when cells experience mechanical stress [24,41]. Activated FAK subsequently phosphorylates Src, which in turn phosphorylates other tyrosine sites on FAK to initiate downstream signaling that results in increased actin polymerization, cell contractility and migration [42]. One of the downstream pathways of FAK is the Rho/ROCK pathway [24,41], which appeared to be inactive in the absence of MMP9. MLC2 is directly downstream of Rho/ROCK signaling, and phosphorylated MLC2 implicates cell contractility by interacting with the actin cytoskeleton [24,41]. Phosphorylated MLC2 was not observed in TG:JNJ or JNJ rat LECs (Figure 8) and, similarly, the protein array showed no notable upregulations of MLC2 or phosphorylated MLC2 between un-MMP9KO and MMP9KO-TG mouse LECs (Table 1 and Figure 3). The above observations on FAK and MLC2 activation suggest that MMP9 may also have a role in regulating integrin-mediated mechanotransduction.

MRTF-A, a downstream target of Rho/ROCK signaling, is a master regulator of TGF-β-induced fibrosis [43,44,45]. Along with SRF, nuclear MRTF-A has been implicated in cytoskeletal remodeling during TGF-β-induced EMT and fibrosis [43,44,45,46,47]. Our laboratory and others have shown that endogenous MRTF-A is localized to the cytoplasm and associated with monomeric G-actin [17,43,44,45]. However, upon TGF-β stimulation, the upregulation of the Rho/ROCK pathway prompts for a greater supply of G-actin for F-actin and αSMA stress fiber formation, and thus, increases G-actin dissociation from MRTF-A [43,44]. Once dissociated from G-actin, MRTF-A translocates to the nucleus, where it acts as a master regulator of TGF-β-induced EMT by upregulating genes associated with myofibroblasts, including MMP9 [43,44]. In the present study, we observed that the nuclear localization of MRTF-A was notably reduced in TG:JNJ in comparison to TG rat LECs (Figure 9). The reduction of nuclear MRTF-A suggests a role for MMP9 in regulating MRTF-A translocation during TGF-β induced EMT.

In conclusion, we have demonstrated a lack of cortactin upregulation, FAK activation and MLC phosphorylation post-TGF-β treatment in our MMP9-deficient models. Since these proteins are involved in actin polymerization and stabilization, and cell migration and contractility, MMP9 appears necessary in regulating TGF-β-induced actin polymerization and cytoskeletal remodeling via these proteins. One pathway that is central to actin polymerization and cytoskeletal remodeling is the Rho/ROCK pathway, which is activated downstream of FAK, and ROCK directly phosphorylates MLC. The reduction in nuclear MRTF-A in TG:JNJ LECs suggests a role for MMP9 in regulating Rho/ROCK/MRTF-A signaling. Our present, and earlier findings clearly indicate that the loss of MMP9 expression or function prevents TGF-β induced EMT in the lens. The current study indicates that MMP9 acts through cytoskeletal signaling to regulate EMT. However, further studies are warranted to fully understand the role of MMP9 in differentially regulating proteins involved in cytoskeletal remodeling, and MMP9 dependence of other TGF-β-induced pathways during TGF-β induced EMT and lens fibrosis.

## 4. Materials and Methods

### 4.1. Reagents

Recombinant human TGF-β2 (TGF-β) was purchased from R&D Systems (Minneapolis, MN, USA). MMP9-specific inhibitor JNJ0966 was purchased from Tocris (Bristol, UK). Primary antibodies that were used include αSMA conjugated to fluorescein isothiocyanate (FITC) from Sigma Aldrich (Oakville, ON, Canada), FAK from Abcam (Waltham, MA, USA), E-cadherin, pFAK at Tyr397, LIMK1, pLIMK1 at Thr508 and cortactin from Invitrogen (Waltham, MA, USA), pMLC2 at Ser18 from Millipore Sigma (Burlington, MA, USA), and MRTF-A from Santa Cruz Biotechnology (Dallas, TX, USA). Secondary antibodies for immunofluorence staining were obtained from Molecular probes (Invitrogen, Carlsbad, CA, USA), and phalloidin conjugated to Alex Fluor^®^568 was obtained from Life Technologies (Eugene, OR, USA).

### 4.2. Obtaining and Culturing LEC Explants

All animal studies were performed according to the Canadian Council on Animal Care Guidelines and approved by McMaster’s Animals Research Ethics Board. Serum-free M199 media was supplemented with penicillin-streptomycin, Amphotericin B and gentamicin for culturing the explants; all of the above reagents were purchased from Gibco by Life Technologies (Gaithersburg, MD, USA). 17–19 days old rat or 21–28 days old mice pups were euthanized using carbon dioxide (CO_2_) followed by cervical dislocation. Whole eyes were removed using curved scissors and placed in a 35 mm polystyrene tissue culture dish containing prewarmed (37 °C) media. The posterior of the eye was located, and the lenses were removed by gently tearing open the eye from the posterior side, and all other ocular structures were discarded. The lenses were transferred to new culture dishes containing fresh and prewarmed (37 °C) media, and the LEC explant containing the intact anterior capsule with LECs was isolated by peeling open the capsule from the posterior end and discarding the fiber mass. The explant was then pinned down using a blunt tool with the LECs facing up to be bathed by the medium. The explants were incubated at 37 °C at 5% CO_2_ and 95% humidity overnight and examined for viability the next day.

### 4.3. Treatments of LEC Explants with TGF-β and JNJ0966

Wildtype and MMP9KO mouse LEC explants were treated with 500 pg/mL of TGF-β or left untreated for 72 h in 2 mL of media.

Rat LEC explants were treated with <0.5% of DMSO, 6 ng/mL or 10 ng/mL of TGF-β for 48 h, 20 µM of JNJ0966 for 48 h, or pretreated for 2 h with 20 µM JNJ0966 and then treated with 6 ng/mL or 10 ng/mL of recombinant human TGF-β for 48 h in 2 mL of media.

### 4.4. Nanostring

Wildtype (WT) (*n* = 4 experiments, where *n* = 3 lenses per experiment), TGF-β overexpressing transgenic (TGFβtg) (*n* = 4 experiments, where *n* = 3 lenses per experiment) or TGFβtg mice on the MMP9KO background (TG:MMP9KO) (*n* = 4 experiments, where *n* = 3 lenses per experiment) at 1.5–2 months of age were sacrificed and their eyes removed. RNA was isolated from the extracted lenses and expression profiling was completed using a 184-gene probe-set custom-designed array on the NanoString nCounter gene expression system, which captures and counts individual mRNA transcripts. The nSolver software was used to normalize the data to the total RNA count, and the ratios of mRNA expression were calculated using the normalized data where one set of WT was used as the reference. Microsoft Excel was used to average the normalized mRNA expression ratios and calculate the standard deviations and *p*-values.

### 4.5. Cytoskeletal Protein Array Using Mouse LEC Explants

An equal number of explants were obtained from male and female mice of each genotype (wildtype or MMP9KO), and the explants were then treated with TGF-β or left untreated for 48 h. Following treatment, protein was harvested for cytoskeletal protein array analyses (*n* = 3 experiments, 10 g of protein per treatment was used for each experiment) (Fullmoon Biosystem, San Francisco, CA, USA). The protein array is focused on proteins involved in actin polymerization and provides the expression of total protein and its active counterparts in the system. The protein expression level was normalized to the median GAPDH signal, and the average normalized protein expression level was calculated using Microsoft Excel. The comparative ratio of proteins from the TGF-β treated wildtype (TG) explants versus untreated (control), and TGF-β treated (MMP9KO-TG) and untreated MMP9KO (un-MMP9KO) explants were calculated using Microsoft Excel and compared. A two-way ANOVA with multiple comparisons was performed and a graph showing data values with standard deviation and *p*-values was plotted using Graphpad Prism software. The graph was further processed using Adobe Photoshop.

### 4.6. Immunofluorescence Staining

Explants were fixed following treatment using 4% paraformaldehyde (PFA) at room temperature for 10–12 min and washed using phosphate buffered saline (PBS). Explants were then lifted from the plates and transferred to separate glass test tubes with PBS. PBS was then removed, and the explants were incubated with permeabilizer (0.1% Triton X-100, 0.5% sodium dodecyl sulphate in PBS) and blocked with 5% normal donkey serum (NDS; Invitrogen, Carlsbad, CA, USA) for 1 h at room temperature. Explants are then incubated with primary antibodies at 1:200 dilution overnight at 4 °C. Explants were washed three times with PBS for 10 min per wash and incubated with secondary antibodies for 1 h at room temperature with gentle rocking. Primary antibodies included phalloidin conjugated to Alex Fluor^®^568, αSMA conjugated to FITC (Sigma Aldrich; Oakville, ON, Canada), FAK (Abcam, Waltham, MA, USA), E-cadherin, pFAK, LIMK1, pLIMK1 and cortactin (Invitrogen, Waltham, MA, USA), pMLC2 (Millipore Sigma, Burlington, MA, USA) and MRTF-A (Santa Cruz Biotechnology, Dallas, TX, USA). Explants were washed three times for 10 min per wash using PBS and mounted using Prolong Gold antifade reagent with 4′6-diamidino-2-phenylindole (DAPI, Invitrogen, Life Technologies, Carlsbad, CA, USA). Fluorescence was detected using Leica DM6 fluorescence microscope. For imaging, we used TG LECs to set the imaging parameters such as exposure, intensity, background correction and magnification as TGF-β results in increase in expression of the proteins mentioned in the manuscript. After standardizing the parameters and imaging TG LECs, we employed same intensity and conditions to image LECs with other treatment conditions. The LECs treated with secondary antibody were considered as negative controls and were used to confirm the specificity of the antibody. Mean fluorescence intensities were detected using Image J. Manders’ coefficients were detected using the JACop plugin added to Image J. Means, relative means and standard error of the mean (SEM) were calculated using Excel, and one-way ANOVAs and Tukey’s Tests were performed using GraphPad Prism software.

## Figures and Tables

**Table 1:** Table showing fold changes of cytoskeletal protein expression. Cytoskeletal protein array analysis was conducted using untreated wildtype (control), wildtype treated with 500 pg/mL TGF-β for 72 h (TG), untreated MMP9KO (un-MMP9KO) and MMP9KO treated with 500 pg/mL TGF-β for 72 h (MMP9KO-TG) mouse LECs (*n* = 3 independent experiments, where 10 µg of protein per treatment was used for each experiment). Red represents upregulation, green represents downregulation and white indicates that no notable difference was observed between the two compared treatment groups. A darker shade of the color of the box indicates a greater fold difference between the two compared treatment groups.

Antibody List	Fold Change Between Samples			
	TG/Control	MMP9KO-TG/un-MMP9KO	TG /un-MMP9KO	TG /MMP9KO-TG
Cofilin (Ab-S3)	2.27	0.93	1.58	1.69
Cortactin (Ab-Y466)	3.11	0.80	1.96	2.45
FAK (Ab-Y910)	2.34	1.03	1.45	1.41
FAK (Ab-pY861)	1.27	0.71	1.11	1.41
Filamin A (Ab-S2152)	1.59	1.16	1.52	1.31
LIMK1 (Ab-T508)	2.85	1.08	1.07	1.00
LIMK1 (Ab-pT508)	1.26	1.17	1.25	1.07
MLC2 (Ab-S18)	1.74	1.06	1.65	1.56
MLC2 (Ab-pS18)	1.57	0.72	0.69	0.95
Rac1/CDC42 (Ab-S71)	1.28	0.76	1.05	1.38
Rho/Rac guanine nucleotide exchange factor (Ab-pS885)	1.31	1.22	1.25	1.03
VASP (Ab-157)	2.08	1.11	1.35	1.21

## Data Availability

The data presented in this study are available in this article.
